# Therapeutic application of chick early amniotic fluid: effective rescue of acute myocardial ischemic injury by intravenous administration

**DOI:** 10.1186/s13619-022-00110-1

**Published:** 2022-04-01

**Authors:** Baiping Cui, Yufan Zheng, Xiang Gao, Lihong Zhang, Borui Li, Jia Chen, Xinyan Zhou, Mengyuan Cai, Wenrui Sun, Yuting Zhang, Keejong Chang, Jiayi Xu, Fuyin Zhu, Yan Luo, Tao Sun, Jin Qian, Ning Sun

**Affiliations:** 1 Wuxi School of Medicine, Jiangnan University, Wuxi, Jiangsu China; 2 Department of Physiology and Pathophysiology, State Key Laboratory of Medical Neurobiology, School of Basic Medical Sciences, Fudan University, Shanghai, 200032 China; 3 Department of Biochemistry and Cancer Institute of the Second Affiliated Hospital, Zhejiang University School of Medicine, Zhejiang, 310009 Hangzhou China; 4 Key Laboratory of Cancer Prevention and Intervention of China National Ministry of Education, Zhejiang, 310009 Hangzhou China; 5 Zhejiang Hygeian Cells BioMedical Co. Ltd, Zhejiang, 310019 Hangzhou China; 6 Stem Cell Application Research Center, the Hangzhou Branch of Yangtze Delta Region Institute of Tsinghua University, Zhejiang, 310019 Hangzhou China; 7 Shanghai Mincal Medical Research Co. Ltd., Large Animal Research Center, Shanghai, 201201 China; 8 Department of Cardiology, Huashan Hospital, Fudan University, Shanghai, 200032 China; 9 Department of Internal Medicine, Huashan Hospital West Campus, Fudan University, Shanghai, 200032 China

**Keywords:** Myocardial ischemic injury, Chick early amniotic fluid, Heart, Yap

## Abstract

Myocardial regeneration has been considered a promising option for the treatment of adult myocardial injuries. Previously, a chick early amniotic fluid (ceAF) preparation was shown to contain growth-related factors that promoted embryonic growth and cellular proliferation, though the nature of the components within ceAF were not fully defined. Here we tested whether this ceAF preparation is similarly effective in the promotion of myocardial regeneration, which could provide an alternative therapeutic for intervening myocardial injury. In this study, a myocardial ischemic injury model was established in adult mice and pigs by multiple research entities, and we were able to show that ceAF can efficiently rescue damaged cardiac tissues and markedly improve cardiac function in both experimental models through intravenous administration. ceAF administration increased cell proliferation and improved angiogenesis, likely via down-regulation of Hippo-YAP signaling. Our data suggest that ceAF administration can effectively rescue ischemic heart injury, providing the key functional information for the further development of ceAF for use in attenuating myocardial injury.

**Supplementary Information:**

The online version contains supplementary material available at 10.1186/s13619-022-00110-1.

## Background

Ischemic heart disease remains the leading cause of human death worldwide (Heusch et al. 2014). In adult mammals, cardiomyocyte proliferation capacity is very limited, which is insufficient to replenish dead cardiomyocytes and recover heart function after myocardial damage (Porrello et al. 2011; Senyo et al. 2013; Shiba et al. 2016). Promoting cardiomyocyte proliferative activity has been considered a potential therapeutic approach for injured hearts. Several developmental and signaling pathways have been shown to stimulate cell cycle re-entry in mature cardiomyocytes and various strategies have been used to target these pathways to stimulate cardiomyocyte renewal in animal models (Mohamed et al. 2018; Magadum et al. 2020; Han et al. 2021; Wu et al. 2021).

Hypoxia, activation of the Hippo-YAP (Yes-associated protein) signaling pathway, and induction of cytokinesis in adult post-mitotic cells by a combination of cell-cycle regulators can significantly improve cardiac function after myocardial infarction (MI) (Cheng et al. 2017; Leach et al. 2017; Nakada et al. 2017; Mohamed et al. 2018). A 2019 study reported that expression of human miR-199a in infarcted pig hearts can stimulate cardiomyocyte proliferation and cardiac repair, although the pigs died 8 weeks later (Gabisonia et al. 2019). These studies suggest that stimulation of endogenous cardiomyocyte proliferation might mediate cardiac repair in adult mammals through different routes. Recent studies also demonstrated that cardiomyocyte proliferation and cardiac regeneration in adult mice correlate with enhanced expression of miR-708 and Pkm2 (Deng et al. 2017; Magadum et al. 2020), which is higher in embryonic and neonatal hearts, and administration of the neonatal extracellular matrix protein Agrin promoted cardiac regeneration and loss of the RNAs that are lower in neonates compared to adults (Bassat et al. 2017). These discoveries suggest that certain factors from the embryonic or neonatal period promote reentry of dormant cardiomyocytes into cell cycle and improve cardiac regeneration.

Amniotic fluid (AF) is the environment where the embryo grows. It serves as a reservoir of fluid and nutrients for the embryos and provides essential growth factors to allow normal development and growth (El-Farrash et al. 2019; Vrachnis et al. 2021). AF includes electrolytes, proteins, hormones, water, carbohydrates, urea, phospholipids, enzymes, and growth factors (Burdett et al. 1982). While in early embryonic development, AF mainly comes from the egg per se which also serves the function of delivering nutrition similar to blood (Larsen 1993). Chick early amniotic fluid (ceAF) can support the development of two-cell mouse embryos (Esmaili and Rezazadeh Valojerdi 2004) and is important for fetal health (Gitlin et al. 1972; Pitkin and Reynolds 1975). Additionally, ceAF can be a source or supplement of cell culture medium (Larsen 1993; Wang et al. 1997). Previous reports have shown that ceAF contains a variety of growth-related factors like nerve growth factor (NGF), transforming growth factor-β (TGF-β), vascular endothelial growth factor (VEGF), insulin-like growth factor (IGF)-I, and IGF-II (tenBusch et al. 1997; Karcher et al. 2005a, 2005b; Mashayekhi et al. 2011; Pressman et al. 2011).

In this study, we test whether ceAF can promote heart repair after ischemic injury. We are able to show that, by multiple independent research entities, the intravenous injection of cell-free ceAF extracted from 7-day-old chick embryos is able to efficiently rescue the damaged cardiac tissues and significantly improve heart function in adult mice and swine models of myocardial ischemic injury. Although ceAF contains multiple components that make it very difficult to determine the single effective therapeutic components at present, our data show, with properties of marked therapeutic effect, little immunogenicity, and easy handling, that ceAF can be further developed to be a novel and safe non-invasive therapy for ischemic heart disease.

## Results

### Intravenous administration of ceAF significantly repaired ischemic heart injury in mouse models

**Fig. 1:**
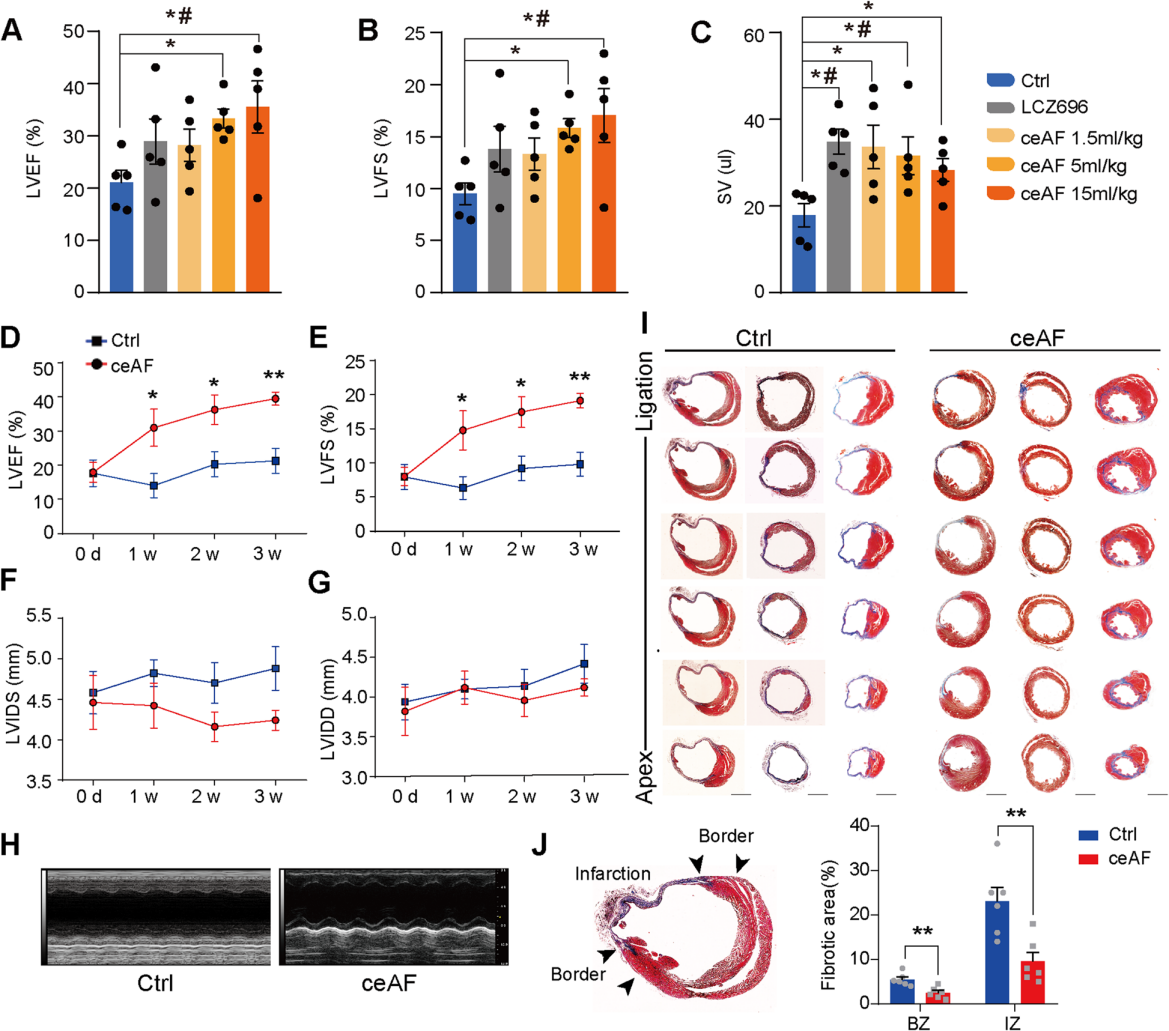
Cardiac Functions and Scar Sizes Following Myocardial Infarction with ceAF Treatment in Mice. **a-c** Cardiac contractile function with ceAF treatment for 12 days after MI in mice. The experimental groups are: i.v. injection of 5% glucose (Ctrl), intragastric administration of LCZ696 at 60 mg/kg (LCZ696), i.v. injection of ceAF at 1.5 ml/kg (ceAF low), 5.0 ml/kg (ceAF middle), and 15.0 ml/kg (ceAF high). Data are mean ± SEM.; *p < 0.05 versus control by t-test; ^#^*p* < 0.05 versus control by one-way ANOVA (Dunnett’s test); *n* = 5 for each group. **d-g** Further evaluation of ceAF treatment in mice with MI. Compared to i.v. injection of 5% glucose (Ctrl), ceAF (5.0 ml/kg) treatment improved LVEF (**d**) and LVFS (**e**) up to 3 weeks post ceAF treatment. **h** Representative echocardiographic images. **i** Masson’s trichrome staining of serial sections of hearts from MI mice 3 weeks post treatment. **j** Scar boundaries are indicated by arrows. Quantification of fibrotic area relative to myocardium in trichrome staining sections demonstrate a significant decrease in scar size in ceAF treated MI mice. Scale bar, 2 mm. Data are mean ± SEM. *, *p* < 0.05; **, *p* < 0.01. (*n* = 6)

Extraction of ceAF is illustrated in Fig. S1. The therapeutic effect of ceAF on myocardial ischemic injury was first examined using mouse left anterior descending coronary artery (LAD) ligation models (Fig. 1a-c and Fig. S2A-J) conducted by Wuxi AppTec (Shanghai). LCZ696, a first-in-class drug for heart failure (Khder et al. 2017), was used as a positive control. There is a trend toward improved left ventricular ejection fraction (LVEF) (Fig. 1a) and left ventricular fractional shortening (LVFS) (Fig. 1b) in LCZ696 treated MI mice compared to the control group. Stroke volumes (SV) (Fig. 1c) and cardiac output (Fig. S2C) were boosted significantly in LCZ696 treated groups (*n* = 5, *p* < 0.05). Treatment with 5.0 ml/kg and 15.0 ml/kg ceAF significantly enhanced LVEF (Fig. 1a) and LVFS (Fig. 1b) in MI mice compared with those in the control group (*n* = 5, *p <* 0.05). Stroke volumes (SV) (Fig. 1c) and cardiac output (Fig. S2C) were also boosted significantly in all three ceAF treated groups (*n* = 5, *p <* 0.05). These results showed a dose dependent cardiac function improvement and the effects of 5.0 ml/kg and 15 ml/kg ceAF treatments were better than LCZ696 treatment on MI. The effects of ceAF treatment on MI using the mouse LAD ligation model were examined further (Fig. 1d-j). Since 5.0 ml/kg ceAF displayed an effective therapy on improving heart function after MI and an intravenous dose of 5.0 ml/kg is suitable for long-term administration in mice, this dose was selected for subsequent experiments. LVEF (Fig. 1d) and LVFS (Fig. 1e) were boosted significantly from the first week to week 3 after ceAF (5.0 ml/kg) treatment compared with those of the control (5% glucose) group (*n* = 6, *p* < 0.05). Both left ventricular internal systolic diameter (LVIDS) and left ventricular internal diastolic diameter (LVIDD) were reduced in value although the numbers were statistically insignificant (Fig. 1f-g). Echocardiographic images displayed that ceAF treatment enhanced the cardiac contractility of MI mice (Fig. 1h). Masson’s trichrome staining of serial heart sections at 3 weeks after MI disclosed more myocardium and smaller infarct in the ceAF treated group (*n* = 3, *p* < 0.01 (Fig. 1i-j).

**Fig. 2:**
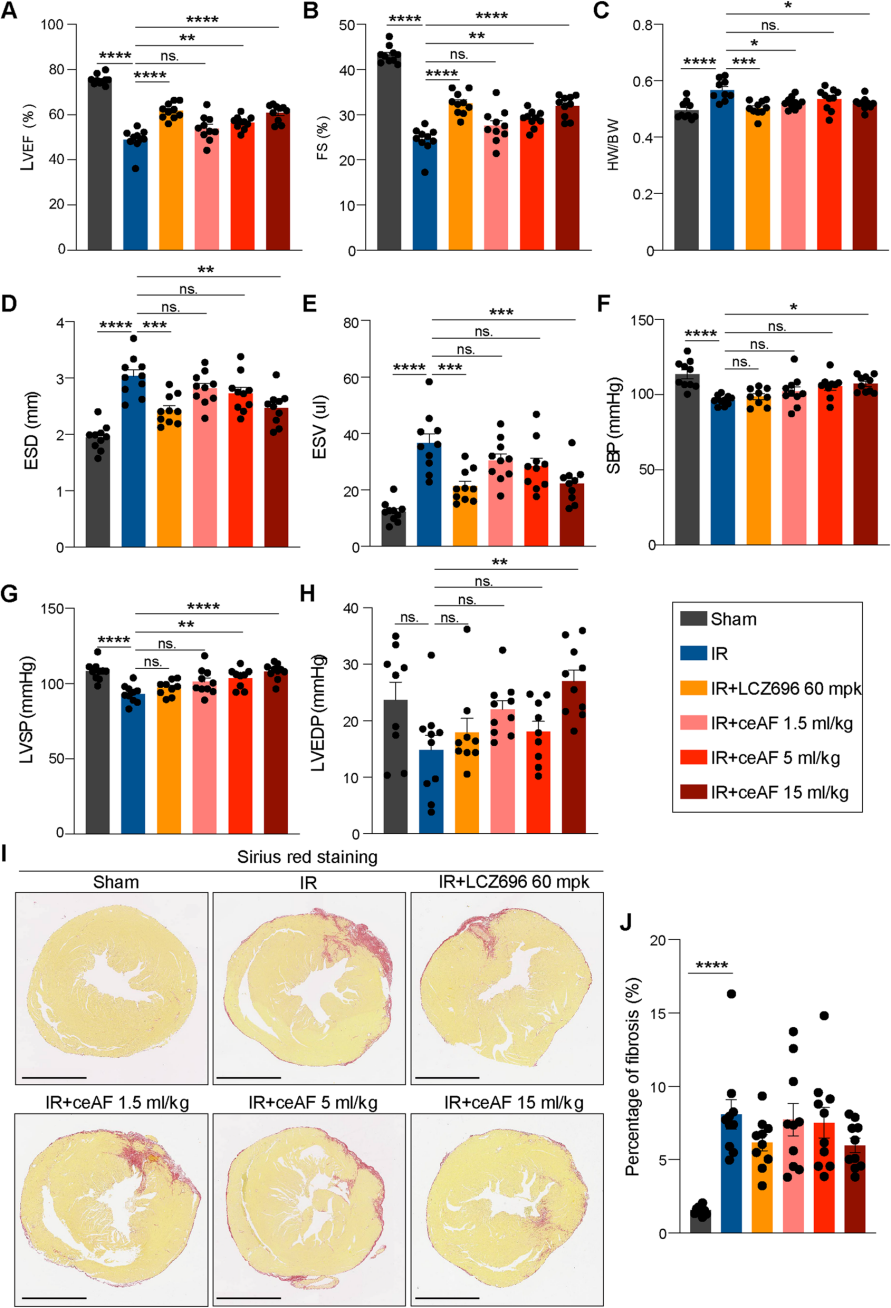
Intravenous Administration of ceAF Improved Cardiac Function in a Mouse Model of Heart Ischemic Reperfusion. Mice with heart IR were treated with ceAF of different doses (1.5 ml, 5 ml, and 10 ml/kg) and LCZ696 (60 mg/kg) as positive control. The LVEF (**a**), FS (**b**), HW/BW (**c**), ESD (**d**), ESV (**e**), SBP (**f**), LVSP (**g**), and LVEDP (**h**) of these mice were improved after 28-day ceAF treatment. **i** Representative images of Sirius red staining on heart tissue sections. Bar, 2 mm. **j** Percentage of fibrosis evaluated according to the Sirius red staining. Data are shown as mean ± SEM. (*n* = 10, ns, no significance, *p < 0.05, **p < 0.01, ***p < 0.001, and ****p < 0.0001 by one-way ANOVA adjusted by Brown-Forsythe test)

Next, the effect of ceAF on heart ischemic reperfusion (IR) injury was examined on C57BL/6 mice at Wuxi AppTec (Shanghai). Similar to the positive control LCZ696, treatment with 5.0 ml/kg and 15.0 ml/kg ceAF significantly enhanced LVEF (Fig. 2a) and LVFS (Fig. 2b) in IR mice compared with those in the IR control group (*n* = 10, *p* < 0.05). Treatment with 15.0 ml/kg ceAF showed the most significant enhancement of heart function and reduction in fibrosis area in IR mice compared with those in the control group (*n* = 10) (Fig. 2). We also tested the effect of 2 weeks administration of ceAF on heart morphology and fibrosis in normal mice under physiological conditions. ceAF had no significant effect on heart weight/body weight ratio (Fig. S3a, b) and no obvious harmful effect on cardiac function of normal mice (Fig. S3c, d). Moreover, Masson’s trichrome staining also indicated that ceAF administration did not induce myocardial fibrosis (Fig. S3e).

### Intravenous administration of ceAF significantly rescued ischemic heart injury in swine IR models

**Fig. 3:**
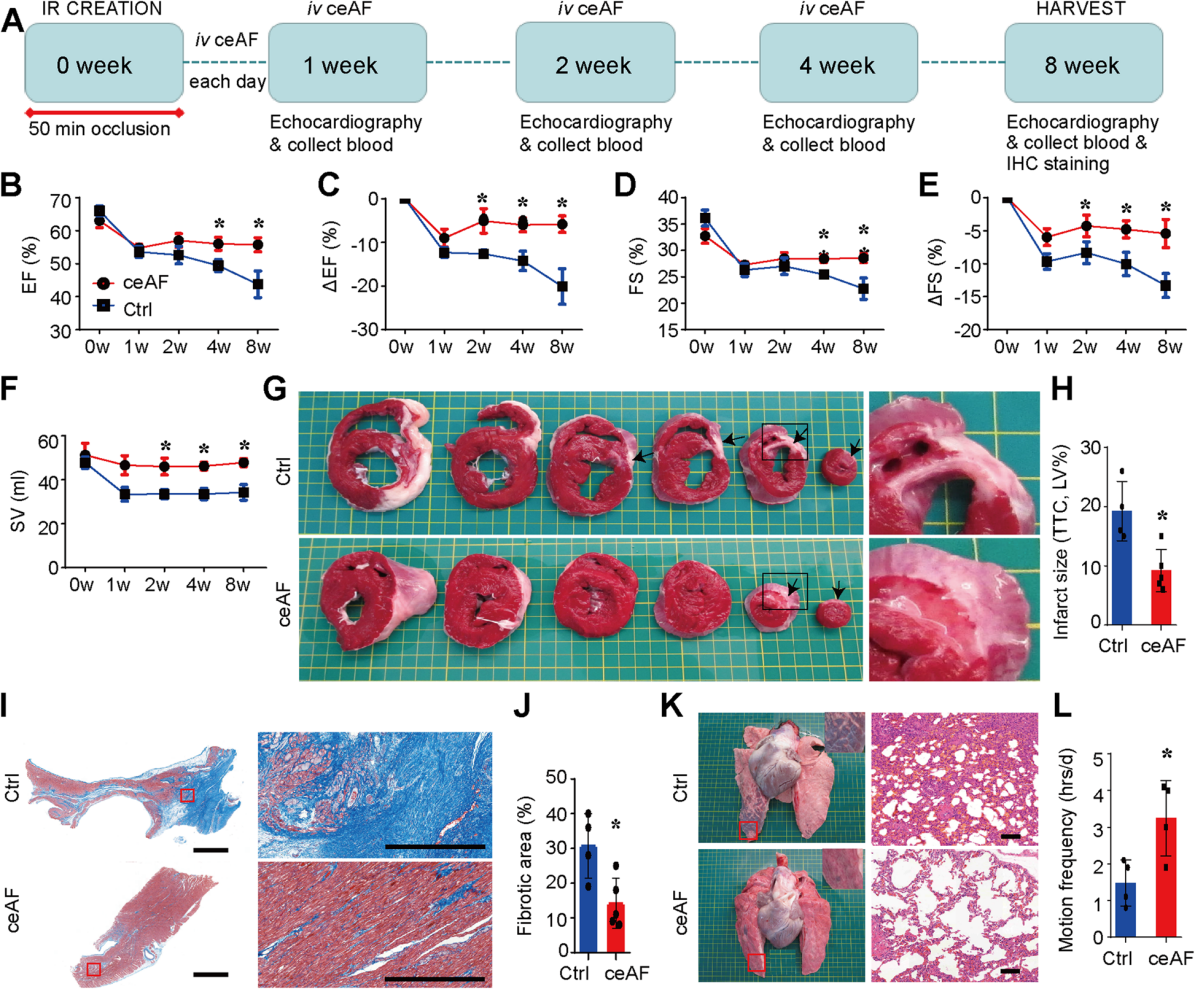
Cardiac Functions and Infarct Sizes with ceAF Treatment in a Swine Model of Ischemia-Reperfusion. **a** Scheme for ceAF treatment on swine model of IR. **b-j** Cardiac functions of pigs with IR and ceAF treatment. LVEF (**b**), at baseline, 1 week, 2 weeks, 4 weeks, and 8 weeks after MI. Change of LVEF (ΔLVEF) (**c**), LVFS (**d**), and change of LVFS (ΔLVFS) (**e**) from 0 week to 8 weeks after IR. **g** Triphenyltetrazolium chloride (TTC) viability staining of heart sections. **h** Quantification of infarct size as a percentage of the left ventricle. **i, j** Representative images showing Masson’s trichrome staining of transverse heart sections from ceAF-treated and control pig hearts 2 months after IR (**i**) with relative quantification of the area of fibrosis in the border zone (**j**). Quantification of fibrotic area was from at least four different regions around the center of infarction of each heart. **k** Representative images showing pulmonary edema in IR pigs with ceAF treatment for 8 weeks compared with control IR pigs treated with glucose solution. **l** Quantification of motion frequency of animals in ceAF- and control- treatment groups within the first week of IR. All data are presented as mean ± SEM, * *p* < 0.05, Student’s two-sided t-test, *n* = 4 for Ctrl, *n* = 5 for ceAF treatment

The therapeutic efficacy of ceAF on myocardial ischemia using swine models of IR was assessed at Shanghai Mincal Medical Research Co. Ltd. We used 1.0 ml/kg of ceAF for the swine model studies, which was roughly equivalent to 5.0 ml/kg in mice as calculated according to previously described guidelines for dosage conversion between experimental animals (Fig. 3a and Fig. S4) (Nair and Jacob 2016). Echocardiographic evaluation on week 0, 1, 2, 4, and 8 indicated that ceAF treatment considerably preserved LVEF and LVFS after IR (Fig. 3b-e). ceAF-treated animals presented with higher stroke volumes (Fig. 3f) and had a trend of reduced LV end-systolic volume (Fig. S5A) and LV end-diastolic volume (Fig. S5B) compared with those of the control group (i.v. 5% glucose) (*n* = 4 for control group, *n* = 5 for ceAF group, respectively). Triphenyltetrazolium chloride (TTC)-staining revealed that infarct size was significantly smaller in the ceAF-treated animals than in the control group (*p* < 0.05) (Fig. 3g-h). Histological studies also verified significantly less fibrosis and more myocardium in the hearts of ceAF-treated pigs (*p <* 0.05) (Fig. 3i-j). Pulmonary congestion and edema were apparent in control animals, which were consistent with the observed symptoms of cough and less daily activity after IR but absent in ceAF-treated pigs (Fig. 3k-l). Animals were euthanized and underwent full necropsy at 8 weeks post-surgery. None showed any macro- or microscopic tissue damage, inflammation, or tumor formation in all non-heart organs examined including liver, kidney, spleen, and lymph node (Fig. S5E-H). Furthermore, routine blood tests (the complete blood count) exhibited no apparent abnormality in pigs after 8 weeks treatment with ceAF or 5% glucose (Table S1), suggesting that ceAF treatment was non-toxic and free of immunogenicity in living pigs.

### ceAF promotes cardiomyocyte proliferation and reduces cardiomyocyte death in ischemic heart injury

**Fig. 4:**
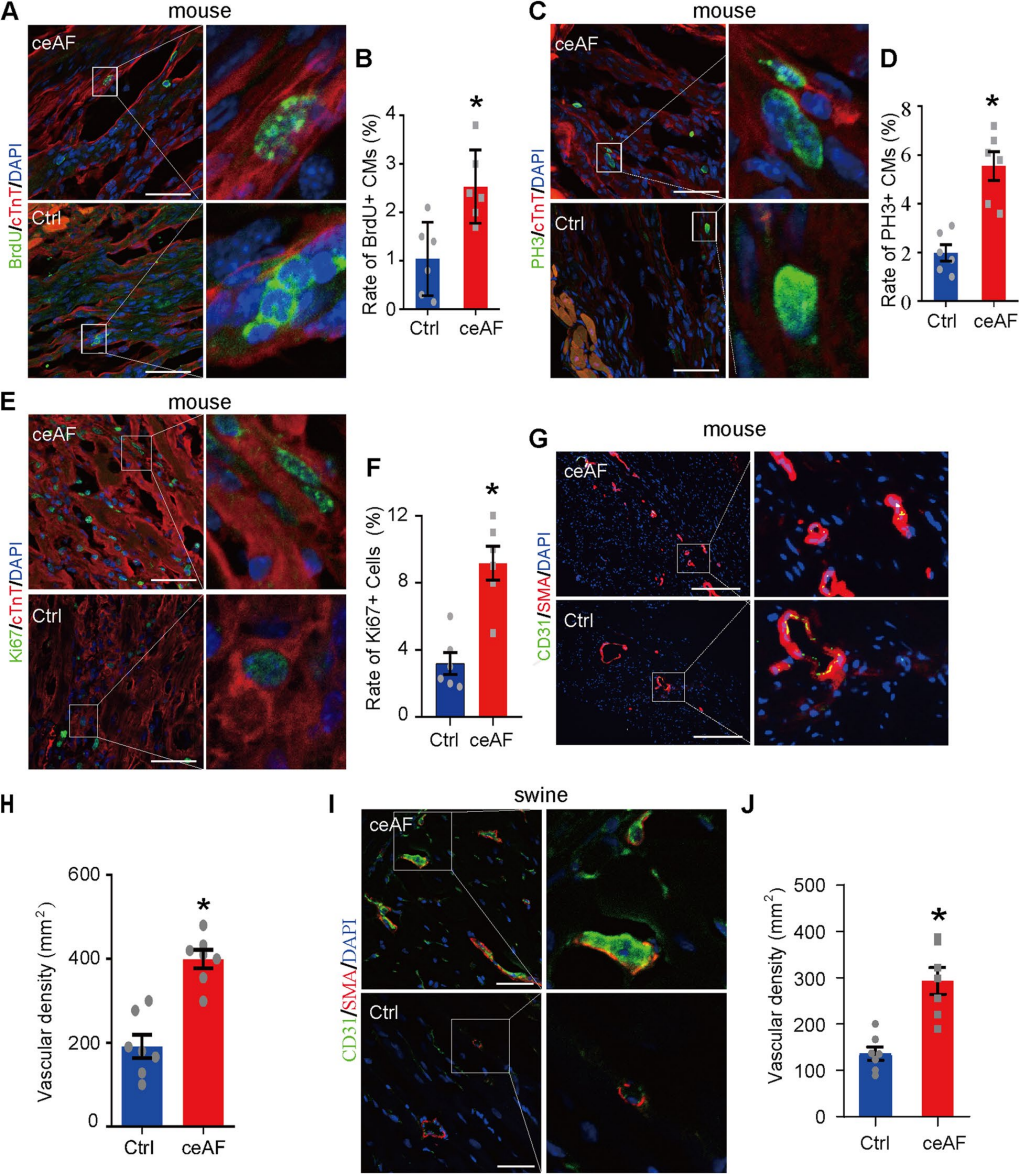
Cardiac cell proliferation and angiogenesis following MI with ceAF treatment. **a** BrdU (green) incorporation in cardiomyocytes from ceAF treated MI mice (*n* = 6 each). The right image shows a higher magnification image of the boxed region in the left image. Scale bars, 100 μm. **b** Quantification of the percent of proliferating cardiomyocytes (BrdU+/cTnT+) in control and ceAF treatment group. *, *p* < 0.05. **c** Co-immunostaining with anti-PH3 (green) and anti-cTnT (red) of heart tissues in the border zone of infarction 7 days after MI. ceAF treatment showed a significant increase in mitosis of cardiomyocytes in MI mice (*n* = 6 each). The right image represents the white boxed area in the left image. Scale bar, 100 μm. **d** Percent of proliferating CMs (PH3+/cTnT+) in response to ceAF treatment. **e** Co-immunostaining with anti-Ki67 (green) and anti-cTnT (red) of heart tissues in the border zone of infarction 7 days after MI (*n* = 6). The right image represents the white boxed area in the left image. Scale bar, 100 μm. **f** Quantification of cells expressing proliferation marker Ki67 in control and ceAF treatment group. *, *p* < 0.05. **g** Co-immunostaining with anti-CD31(green) and anti-smooth muscle actin (red) of heart tissues from MI mice treated with 5% glucose or ceAF (*n* = 4 each). Scale bars, 100 μm. **h** Vascular density in heart sections of control and ceAF treatment group was quantified by calculating the number of the CD31-positive vascular structures. *, *p* < 0.05. **i** Co-immunostaining with anti-CD31 (green) and anti-smooth muscle actin (red) of heart tissues from IR pigs treated with 5% glucose or ceAF (*n* = 4 each). Scale bars, 100 μm. **j** Vascular density in heart sections of control and ceAF treatment group. The density was quantified by calculating the number of the CD31-positive vascular structures. *, *p* < 0.05. Scale bars, 100 μm

To study biological functions of ceAF, mice heart sections were immunostained with the cardiomyocyte-specific marker cTnT together with the cell proliferation marker 5-Bromo-2-deoxyuridine (BrdU) and the mitosis marker phosphorylated histone H3 Ser10 (PH3). In the ceAF treatment group, mice hearts had ~ 2.5% cellular BrdU incorporation at 1 week after MI, which was significantly higher than that in the control mice (~ 1%) (*p* < 0.05) (Fig. 4a and b). The numbers of PH3 positive cardiomyocytes were also significantly increased in hearts from ceAF-treated mice (*p* < 0.05) (Fig. 4c and d). Moreover, Ki67-positive cells significantly increased after ceAF treatment (*p* < 0.05), demonstrating that ceAF increased total cell proliferation activity in the heart after MI (Fig. 4e and f). Moreover, heart sections of the border zone of MI were double stained with the blood vessel marker smooth muscle actin (SMA) and CD31. The border zones of infarction in both adult mice (*p* < 0.05) and pigs (*p* < 0.05) treated with ceAF were inspected, and significant elevations in the number of small blood vessels were observed (Fig. 4g-j). The results of cell proliferation and increased blood vessel formation were consistent with the data of improved cardiac function following MI by ceAF treatment. Death of cardiomyocytes is an essential mechanism of pathological remodeling after MI. Compared to the control groups, using TUNEL (terminal deoxynucleotidyl transferase-mediated deoxyuridine triphosphate nick end labeling) staining, we detected fewer apoptotic cells in the hearts of mice in the ceAF-treated groups 3 and 24 h after artery ligation (*, *p* < 0.05; Fig. S6A, B).

### ceAF downregulates hippo-YAP pathway in cardiomyocytes of injured myocardium

**Fig. 5:**
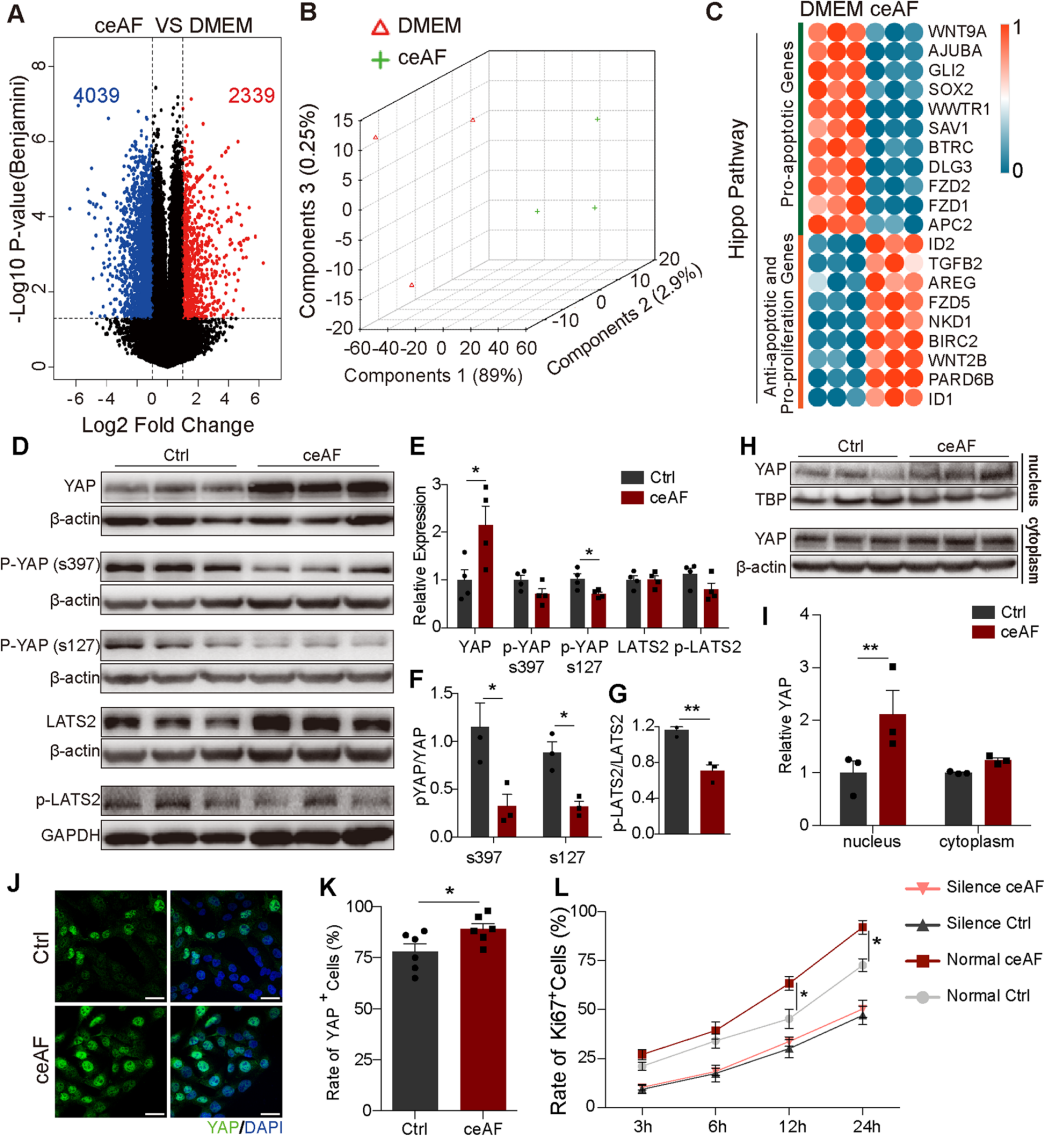
ceAF promotes cardiomyocyte proliferation by inhibiting Hippo-YAP signaling in AC16 cells. The human cardiomyocyte cell line AC16 was used for initial in vitro studies. **a** Volcano plot of the RNA-seq data of ceAF treated versus the control (DMEM) treated AC16 cells (*n* = 3 per group). **b** Principal Component Analyses (PCA) of the total RNA-seq data (*n* = 3 per group). **c** Expression of genes involved in the Hippo pathway after 24 h treatment in AC16 strain. **d** Western blots of YAP, p-YAP S397, p-YAP S127, LATS2, and p-LATS2 in AC16 cells treated with DMEM or ceAF (*n* = 3 per group). **e-g** Quantitative analyses of the immunoblots in (**d**). **h, i** Western blots (**h**) and quantitative analyses (**i**) of intranuclear and cytoplasmic YAP, respectively, in AC16 cells treated with DMEM or ceAF (*n* = 3 per group). **j, k** Representative images and quantitative analyses of immunostaining of anti-YAP (green) and DAPI in AC16 cells treated with DMEM or ceAF (*n* = 6 each). Scale bar, 100 μm. **l** Percent of Ki67 positive cells over time after ceAF treatment in AC 16 cells with or without siRNA silencing of YAP expression. (*n* = 3 each). *, *p* < 0.05, **, *p* < 0.01

To understand the underlying molecular mechanism, we performed whole transcriptome RNA-sequencing in the human cardiomyocyte cell line AC16 treated with DMEM or ceAF for 24 h after fasting to mimic the ischemic animal model (Fig. 5a-c). ceAF significantly reduced expression of pro-apoptotic genes and increased expression of anti-apoptotic and proliferation genes in the Hippo-Yap pathway in AC16 cells (Fig. 5c). ceAF treatment decreased phosphorylation of Yap at serine 397 and serine 127 as well as phosphorylation of LATS2 (Fig. 5d-g). Further, western blot results indicated that ceAF-treatment increased the level of cytoplasmic and nuclear Yap (Fig. 5h-i), suggesting that ceAF-treatment promoted YAP entry into the nucleus. Fluorescence staining results were consistent with western blot results, which also proved that ceAF increased YAP entry into the nucleus (Fig. 5j-k). Inhibiting YAP expression by siRNA significantly diminished the effect of ceAF on cell proliferation (Fig. 5l).

**Fig. 6:**
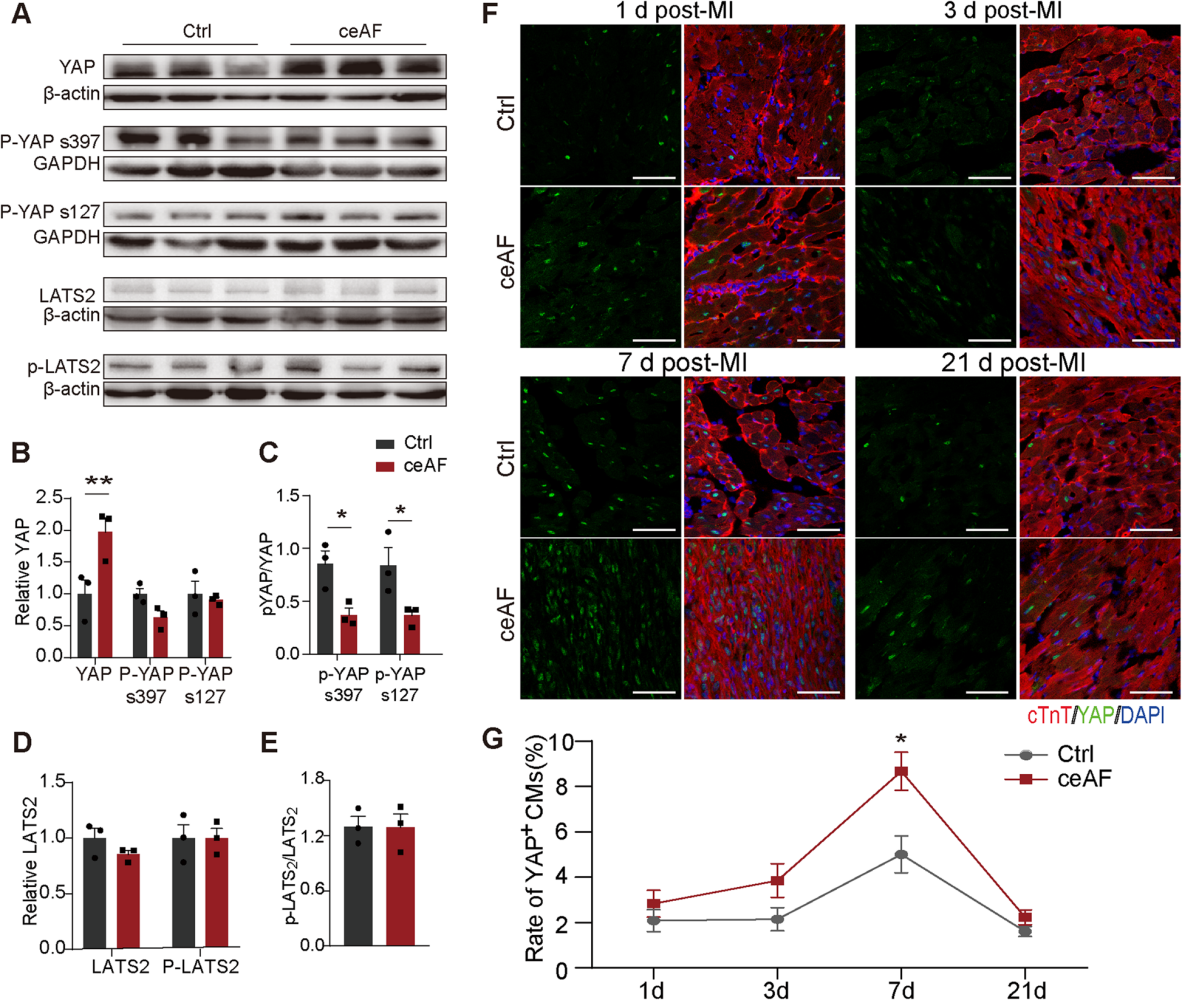
ceAF administration inhibited the Hippo-Yap signaling in MI mice. **a** Western blots of YAP, phosphorylated-YAP at Ser397 (p-YAP, S397), phosphorylated-YAP at Ser127 (p-YAP, S127), LATS2, and phosphorylated-LATS2 (p-LATS2) in MI mice treated with ceAF or 5% glucose (control) (*n* = 3 per group). **b-e** Quantitative analyses of the immunoblots in (A). **f, g** Representative images and quantitative analyses of co-immunostaining with anti-YAP (green) and anti-cTnT (red) of heart tissues in the border zone of infarction 1 day, 3 days, 7 days, and 21 days after MI. (*n* = 4 each). Scale bar, 100 μm. *, *p* < 0.05

We evaluated the effect of ceAF on the Hippo-YAP pathway in mice with MI based on in vitro data that ceAF treatment significantly down-regulated Hippo-YAP signaling in AC16 human cardiac cells (Fig. 6). Compared with controls, western blots on heart samples of ceAF treated MI mice revealed that the overall protein level of YAP was significantly increased (Fig. 6a-b). Although phosphorylation of LATS2 was not changed much, pYAP/YAP at serine 397 and serine 127 were significantly lower than those of the controls (*p* < 0.05) (Fig. 6b-e). Nuclear staining of YAP was also significantly increased in the first week and gradually returned to the basal level by week 3 (Fig. 6f-g).

The Hippo-YAP pathway has great potential for therapeutic manipulation in cardiac disease states and to trigger endogenous heart regeneration. Our results suggest that the biological effect of ceAF to injured heart is likely via the downregulation of the Hippo-YAP pathway, which may effectively promote the regenerative potential and improve angiogenesis in the injured myocardia.

## Discussion

In this study, we have shown that intravenous administration of ceAF significantly improved cardiac function after acute myocardial ischemic injury both in adult mice and pigs. ceAF markedly enhanced cardiac systolic capacity and reduced fibrotic scar size in MI mice, similar to or exceeding the therapeutic results of the LCZ696 treatment. ceAF also prominently improved heart function, alleviated pulmonary congestion, and increased daily activities after IR injury in a large swine model. These data from multiple independent research entities all indicate that ceAF is very effective in the rescue of acute myocardial ischemic injury.

Since the initial discovery of the Hippo–YAP pathway in genetic screens in *Drosophila melanogaster*, this pathway has emerged as an important regulator of tissue renewal. Our data suggest that the therapeutic functions of ceAF for acute myocardial ischemic injury were achieved through inhibiting Hippo-YAP signaling to improve cardiomyocyte regeneration. This therapeutic mechanism matches the mechanism reported by Ronald J. Vagnozzi (Bian et al. 2019, Aziz et al. 2020). ceAF is a natural product in which the concentration of any factor may be higher than in adult tissues but is still within a biological range which is usually just slightly lower than a therapeutic dosage. It is believed that the ceAF functions through low levels of many factors working synergistically.

In this study, choosing 7 day-AF is a comprehensive consideration of industrialization and scientific research, as there is less yield from each fertilized egg before 7 days and the protein content is increased day by day in > 8 day- amniotic fluid (Karcher et al. 2005a, 2005b; Da Silva et al. 2017; Da Silva et al. 2019). We also found that ceAF contains 10 fractions through HPLC, and further cell experiments indicated that peak 6 (P6) and peak 8 (P8) fractions could significantly promote cell proliferation on the cardiomyocyte line AC16. Further functional evaluation of P6 and P8 components showed that they both have a therapeutic effect on myocardial infarction mice (data not shown), however, the therapeutic effect is less than that of ceAF. The multiple components of ceAF and its effective therapeutic components for heart repair are worth further clarification in the future.

## Conclusion

Our data indicates that intravenous injection of ceAF effectively rescued ischemic heart injury through modulation of the Hippo-Yap pathway. Our findings also point to a substantial translational potential of ceAF to be a novel and safe non-invasive therapy for ischemic heart disease in the future.

## Methods

### Animals

 All experiments were performed on age-matched male mice. C57BL/6 J mice were purchased either from Shanghai Slake Laboratory Animal Co. LTD. (Shanghai, China) or ZheJiang Vital River Laboratory Animal Technology Co. Ltd. (JiaXing, ZheJiang, China). Pigs (large white) were supplied by Shanghai Qidong Longning Science and Technology Agricultural Development Co. LTD. (Shanghai, China).

### Preparation and separation of ceAF

Fertilized chick (*Gallus gallus* domesticus) eggs were purchased from Hangzhou Xiaoshan Xintang Yangji Farm (Hangzhou, Zhejiang, China) and were incubated at 38 ± 1 °C with a humidity of 50%. ceAF was collected from day 7 according to development stage based on Hamburger and Hamilton (Yang et al. 2003). The aliquots were centrifuged at 2352 g for 20 min and the supernatants were filtered through a 0.22 μm sterile filter (Millipore, China). The samples were stored at − 80 °C for long term storage.

### Mouse model of acute myocardial infarction

Induction of anterior wall MI in 8-week mice (~ 20 g) was carried out using previously described procedures (Cui et al. 2019). Generally, male C57BL/6 J mice were anesthetized by inhalation of isoflurane mixed with oxygen, endotracheally intubated and then ventilated using a volume-controlled ventilator. The heart was exposed by a minimally invasive left thoracotomy and the LAD was ligated permanently for induction of acute MI. Occlusion of the LAD resulted in immediate blanching of the anterior wall of the left ventricle indicative of myocardial ischemia. On day 3, mice with LVEF< 40% were selected for subsequent experiments. LCZ696 was purchased from Beijing Novartis Pharma Co. Ltd. (Beijing, China).

### Mouse model of acute myocardial ischemia-reperfusion (IR)

8-week male C57BL/6 J mice (~ 20 g) were used for model establishment after a week adaption to the environment. The animals were anesthetized with isoflurane (1.5%–5% V/V in oxygen) inhalation, endotracheally intubated and then ventilated using a volume-controlled ventilator. The chest was opened between the third and fourth ribs, and a chest expander was used to expose the heart. The left anterior descending coronary artery was ligated with 8–0 silk thread for 30 min, and then the ligation thread was removed for blood reperfusion.

### Swine model of acute myocardial ischemia-reperfusion

The pigs were subjected to transluminal closed-chest left anterior descending (LAD) coronary artery balloon occlusion (van Hout et al. 2017) for 50 min followed by reperfusion. Study design is shown in Supplementary material online, Fig. S2. Male and female pigs weighing 45–55 kg were anesthetized with ketamine hydrochloride (15 mg/kg, IM). Animals were endotracheally intubated and ventilated using a volume-controlled ventilator with 100% O_2_, supplemented with 2.5% sevoflurane. Animals were fixed in supine position and connected with multiple electrophysiological apparatus. The right or left femoral artery was identified, and a 6-Fr femoral arterial introducer sheath (Terumo, Japan) was advanced over the guidewire in the femoral artery by percutaneous puncture. A three-connected tee plate and ring-handle syringe were connected to contrast agent, heparin normal saline, and invasive blood pressure transducer, respectively. Heparin sodium was given as a 150 IU/kg bolus i.v. at the start of the procedure and continued at 50 IU/kg/h i.v throughout the operation. Femoral artery angiography was performed (Supplementary material online, Fig. S2A), and a 6-Fr guiding catheter was advanced into the ostium of the coronary artery. Baseline selective coronary angiography and left ventricular angiography were performed and the target blocking location was determined. Lidocaine (0.03 mg/kg/min) was intravenously infused throughout the following procedures. A matched angioplasty balloon (Perouse, France) was advanced to the mid-LAD at 10–12 atm to block the blood flow completely. Each time, the balloon was inflated to 10–12 atm for 10 min followed by a 5-min deflated interval. The total balloon occlusion time was 50 min and the corresponding leads of electrocardiography (ECG) stably presented ST-segment elevation and pathological Q wave. Afterwards, the balloon was deflated and detached and thereafter the LAD was reperfused. Finally, the arterial sheath was removed, followed by compression of the femoral artery puncture area for 30 min. After the procedures, we fixed an indwelling needle to an ear vein of the pig by medical tape and the daily injection was carried out through the indwelling needle. All the animals received penicillin 640 WU (Shanghai Nuotai Chemical Co. Ltd., China) once a day for 3 days. In case of ventricular fibrillation (VF), non-synchronized direct current defibrillation was performed at 150 J.

### Fibrotic scar area quantification

Control and treated MI mouse hearts were paraffin-sectioned and processed for standard Masson’s trichrome staining. Fibrotic areas on trichrome stained sections were quantified with Image J. Pigs were anaesthetized and euthanized by injection of 10% KCl to stop the heart at diastole. The excised hearts were sectioned in 1-cm thick slices, starting from the apex towards the base. Slices were incubated in 1% TTC (Sigma, US) 0.9% NaCl at 37 °C for 25 min to discriminate infarcted tissue from viable myocardium. The infarcted area was calculated as percentage of the left ventricle.

### Immunoblot

Proteins were extracted from the myocardial cell line AC16 and heart tissues. Nucleoprotein and plasma protein were prepared using a nuclear extraction kit (Beyotime Biotechnology, China). Equal amounts of samples were separated by SDS-PAGE and transferred onto PVDF membranes. The following primary antibodies were used: YAP (1:1000, #14074, Cell Signaling Technology, US), Phospho-YAP (Ser127) (1:1000, #13008, Cell Signaling Technology, US), LATS2 (1:1000, # 5888, Cell Signaling Technology, US), Phospho-LATS2 (1:1000, #8654, Cell Signaling Technology, US) (1:1000, #13008, Cell Signaling Technology, US). TBP (1:1000, #44059, Cell Signaling Technology, US), TATA binding protein (TBP) is a widely expressed nuclear protein. β-Actin (1:1000, # 3700, Cell Signaling Technology, US), GAPDH (1:1000, # 5174, Cell Signaling Technology, US). The regions containing proteins were visualized by the enhanced chemiluminescence system (ECL Prime Western Blotting Detection Reagent, GE Healthcare, Buckinghamshire, UK). Densitometric analyses were performed with Image J Software (National Institutes of Health, USA).

### Histology and immunofluorescence (IF) staining

Tissues were harvested and fixed in 4% paraformaldehyde (PFA)/PBS solution at room temperature and then processed for either paraffin or cryostat embedding and sectioned. H&E and Masson’s trichrome staining were performed according to standard procedures on paraffin sections. The frozen slides were permeabilized with 0.5% Triton X-100 for 15 min and blocked with normal goat serum for 30 min. Then, samples were incubated with the following antibodies diluted in 3% BSA (Sigma, US) blocking solution and 1% goat serum: Anti-cTnT (1:200, ab47003, Abcam, US) antibodies were used to identify cardiomyocytes. Anti-BrdU (1:200, ab152095, Abcam, US), anti-phosphorylated-histone 3 (PH3) (1:100, 06–570-AF488, Millipore, US) and Ki67 (1:300, ab15580, Abcam, UK or US) antibodies were used to analyze cell cycle re-entry, DNA synthesis and karyokinesis respectively. Other antibodies used in the studies included anti-CD31 (1:200, ab28364, Abcam, US), anti-actin (1:200, AA132, Beyotime, China) and YAP (1:300, #14074, Cell Signaling Technology, US). After three 5-min washes with PBS, samples were stained at room temperature for 1 h with fluorescent secondary antibodies (Abcam, US) followed by 10 min of DAPI staining for nucleus visualization. Slides were viewed under a fluorescence microscope (Olympus live cell imaging microscope, Japan) or spinning-disc confocal microscope (Carl Zeiss, Germany). For the quantification of the numbers of BrdU, PH3, or Aurora B+ cardiomyocytes, the results acquired from at least 3–5 sections of the heart harvested from each animal at the ventricular valve level of the 4-chamber view, or at the level of ligature of the 2-chamber view, with at least 100 μm distance from each other were averaged. In all cell counting experiments, fields of view were randomized to reduce counting bias.

### Statistical analysis

Statistical analysis was performed using GraphPad Prism 9. Experimental data were reported as mean ± SEM. To compare the differences between two groups, two-tailed Student’s t-test was used. Statistical differences among more than two groups were analyzed with one-way analysis of variance (ANOVA) tests. * *p* < 0.05 and * * *p* < 0.01 were considered as statistical significance.

## Supplementary Information

**Additional file 1: Supplemental Fig. 1.** Schematic diagram of Chick Early Amniotic Fluid (ceAF) extraction. **Supplemental Fig. 2.** Analyses of cardiac function parameters before and after treatment of ceAF on mice MI model. a-b Grouping of mice based on the EF (a) and body weight (b) values on the second day after MI. Each group (*n* = 5) showed a very close average value of EF and body weight at this time. c-i Assessment of cardiac output (c), heart rate (d), LV mass (e), systolic left ventricular posterior wall thickness (LVPWTs) (f), diastolic left ventricular posterior wall thickness (LVPWTd) (g), end-diastolic volume (EDV) (h), and end-systolic volume (ESV) (i) of MI mice at day 13 after MI (*n* = 5. One-way ANOVA). j Average body weight values of MI mice from each group during the experiment. Data were mean ± SEM, *p < 0.05. Student’s two-sided t-test was used. **Supplemental Fig. 3.** ceAF has no significant effect on normal heart function. a Macroscopic view of the heart of C57BL/6 J mice treated with or without ceAF. b Quantification of heart weight/body weight ratio in each group, *n* = 6. The LVEF (c) and LVFS (d) values in normal C57BL/6 J mice treated with ceAF (5.0 ml/kg) (Ctrl+ceAF) or with 5% glucose (Ctrl) for 14 days. Data were mean ± SEM. e Representative Masson’s trichrome staining images of heart sections (left, scale bars: 2 mm) and high-magnification views of LV wall (right, scale bars: 50 μm) of each group. f Quantification of fibrosis area relative to myocardium in Masson’s trichrome staining sections. Data are mean ± SEM. ns, non-significant. *n* = 6. **Supplemental Fig. 4.** Establishment of ischemia reperfusion model in pigs. a Pigs were disinfected with iodide at the site of femoral vein puncture after anesthesia. b Coronary angiography was performed using contrast guidewire and the balloon was delivered to the left coronary artery. c LAD-D1 was selected as the infarct site. d The electrocardiogram V1 to V6 leads showed an electrocardiogram with T wave elevation. T wave elevation was documented in all pigs with IR. e Immediately after surgery, cardiac ultrasound was performed to measure cardiac function. **Supplemental Fig. 5.** Additional cardiac function parameters and typical macroscopic and histological appearance of liver, kidney, spleen, and lymph node of MI pigs treated with ceAF after 8 weeks. Compared with those of the control group, ceAF-treated animals presented with a trend toward reduced LV end-systolic volume (LVESV) (a), LV end-diastolic volume (LVEDV) (b), LVIDD (c) and LVIDS (d). e-h Toxicity of ceAF treatment for 8 weeks. There were no obvious differences observed in all organs examined including liver (e), kidney (f), spleen (g), and lymph node (h) in IR pigs compared with Ctrl IR pigs treated with glucose solution (*n* = 4 for Ctrl, *n* = 5 for ceAF treatment). **Supplemental Fig. 6.** ceAF protects mouse cardiomyocytes from ischemia-induced apoptosis. a TUNEL staining of heart tissues in the border zone of infarction 3 h and 24 h after MI. b Percent TUNEL+ nuclei in mouse heart sections after 3 h or 24 h with or without ceAF treatment (*n* ≥ 3 mice), *, *p* < 0.05. Scale bars, 100 mm.

**Additional file 2: Table S1.** Routine blood tests in pigs.

## Data Availability

The datasets used and/or analyzed during the current study are available from the corresponding author on reasonable request.

## Abbreviations

ceAF: Chick early amniotic fluid
AF: Amniotic fluid
MI: Myocardial infarction
IR: Ischemia reperfusion
LAD: Left anterior descending coronary artery
LVEF: Left ventricular ejection fraction
LVFS: Left ventricular fractional shortening
SV: Stroke volumes
LVIDS: Left ventricular internal systolic diameter
LVIDD: left ventricular internal diastolic diameter
TTC: Triphenyltetrazolium chloride
IF: Immunofluorescence
TBP: TATA binding protein
