# Supplementary material for: Therapeutic application of chick early amniotic fluid: effective rescue of acute myocardial ischemic injury by intravenous administration
Source: Cell Regen. 2022 Apr 1;11:9. doi: 10.1186/s13619-022-00110-1 (PMC8975954; doi:10.1186/s13619-022-00110-1)
Supplement: Supplementary file 1 — Additional file 1: Supplemental Fig. 1. Schematic diagram of Chick Early Amniotic Fluid (ceAF) extraction. Supplemental Fig. 2. Analyses of cardiac function parameters before and after treatment of ceAF on mice MI model. a-b Grouping of mice based on the EF (a) and body weight (b) values on the second day after MI. Each group (n = 5) showed a very close average value of EF and body weight at this time. c-i Assessment of cardiac output (c), heart rate (d), LV mass (e), systolic left ventricular posterior wall thickness (LVPWTs) (f), diastolic left ventricular posterior wall thickness (LVPWTd) (g), end-diastolic volume (EDV) (h), and end-systolic volume (ESV) (i) of MI mice at day 13 after MI (n = 5. One-way ANOVA). j Average body weight values of MI mice from each group during the experiment. Data were mean ± SEM, *p < 0.05. Student’s two-sided t-test was used. Supplemental Fig. 3. ceAF has no significant effect on normal heart function. a Macroscopic view of the heart of C57BL/6 J mice treated with or without ceAF. b Quantification of heart weight/body weight ratio in each group, n = 6. The LVEF (c) and LVFS (d) values in normal C57BL/6 J mice treated with ceAF (5.0 ml/kg) (Ctrl+ceAF) or with 5% glucose (Ctrl) for 14 days. Data were mean ± SEM. e Representative Masson’s trichrome staining images of heart sections (left, scale bars: 2 mm) and high-magnification views of LV wall (right, scale bars: 50 μm) of each group. f Quantification of fibrosis area relative to myocardium in Masson’s trichrome staining sections. Data are mean ± SEM. ns, non-significant. n = 6. Supplemental Fig. 4. Establishment of ischemia reperfusion model in pigs. a Pigs were disinfected with iodide at the site of femoral vein puncture after anesthesia. b Coronary angiography was performed using contrast guidewire and the balloon was delivered to the left coronary artery. c LAD-D1 was selected as the infarct site. d The electrocardiogram V1 to V6 leads showed an electrocardiogram with T wave ele [file 13619_2022_110_MOESM1_ESM.docx]

**Supplementary material**

**Therapeutic Application of Chick Early Amniotic Fluid: Effective Rescue of Acute Myocardial Ischemic Injury by** **Intravenous Administration**

Baiping Cui ^1,2^†, Yufan Zheng ^1,2^†, Xiang Gao ^3,4,7,8^†, Lihong Zhang ^3,4,7,8^†, Borui Li ^2^, Jia Chen ^2^, Xinyan Zhou ^2^, Mengyuan Cai ^2^, Wenrui Sun ^2^, Yuting Zhang ^2^, Keejong Chang ^7,8^, Jiayi Xu ^7,8^, Fuyin Zhu ^9^, Yan Luo ^3,4,7,8^*^,^ Tao Sun ^5,6^*, Jin Qian ^7,8^*, Ning Sun ^1,2^*

**Supplementary Methods**

**Echocardiography**

Assessment of in vivo cardiac function on mice was performed with the Vevo 3100 micro-ultrasound system (Visual Sonics, Canada) at baseline at 3 days post-injury. Hearts were imaged in 2D long-axis view at the level of the greatest LV diameter in animals under light general anesthesia. This view was used to position the M-mode cursor perpendicular to the LV anterior and posterior walls. Echocardiography performed 3 days post-MI demonstrated a severe reduction in LVEF. Echocardiography was then performed on day 7, 14, and 21 after treatment. LVEF, LVFS, LVIDD and LVIDS were calculated at all time-points. Cardiac ultrasound was performed on MI mice treated with different doses of ceAF on day 12 and IR mice on day 28 with the Vevo 3100 micro-ultrasound system.

Cardiac ultrasound was performed on pigs on day 1, 7, 14, 28, and 56 after IR with the NemioMX ultrasound system (Toshiba, Japan). Animals were sedated with 4 mg/kg tiletamine hydrochloride injected intramuscularly and light anesthesia was maintained by continuous inhalation of 2.5% sevoflurane. Pigs were placed in a right lateral position. Heart rate and ECG were monitored continuously.

**5-Bromo-2-deoxyuridine (BrdU) Administration**

BrdU (Sigma, US) was dissolved in ddH2O and filtered. To record cell proliferation in vivo, 50 mg/kg BrdU was injected intraperitoneally daily and for 5 days following MI. Hearts were harvested on day 8 and subjected to BrdU immunohistochemistry using an anti-BrdU antibody (ab152095, Abcam, US).

**Cell culture**

The AC16 cell line was acquired from BeNa Culture Collection (BNCC337712, China) and maintained in DMEM with high glucose, glutamine, and sodium pyruvate (BI, Kibbutz Beit-Haemek, Israel) supplemented with 10% FBS (ExCell Bio, China), 100 U/ml penicillin (Keyi, China) and 100 mg/ml streptomycin (Keyi, China). Cells were maintained at 37℃, 5% CO2 until 80% confluence.

**Intravenous injection**

ceAF was administered to mice by tail vein injection. First, the body of the mouse was covered with a special cover for tail vein injection, and only the tail was exposed. After incubating the mouse tail with a warm towel for about 1 min, the blood vessels of the mouse tail became prominent. Then the tail was straightened and injected horizontally with an insulin needle, and blood was allowed to flow back into the needle allowing for injection of ceAF.

Pigs were administered through an indwelling needle placed in the ear vein.

**Supplementary figures and legends**

**
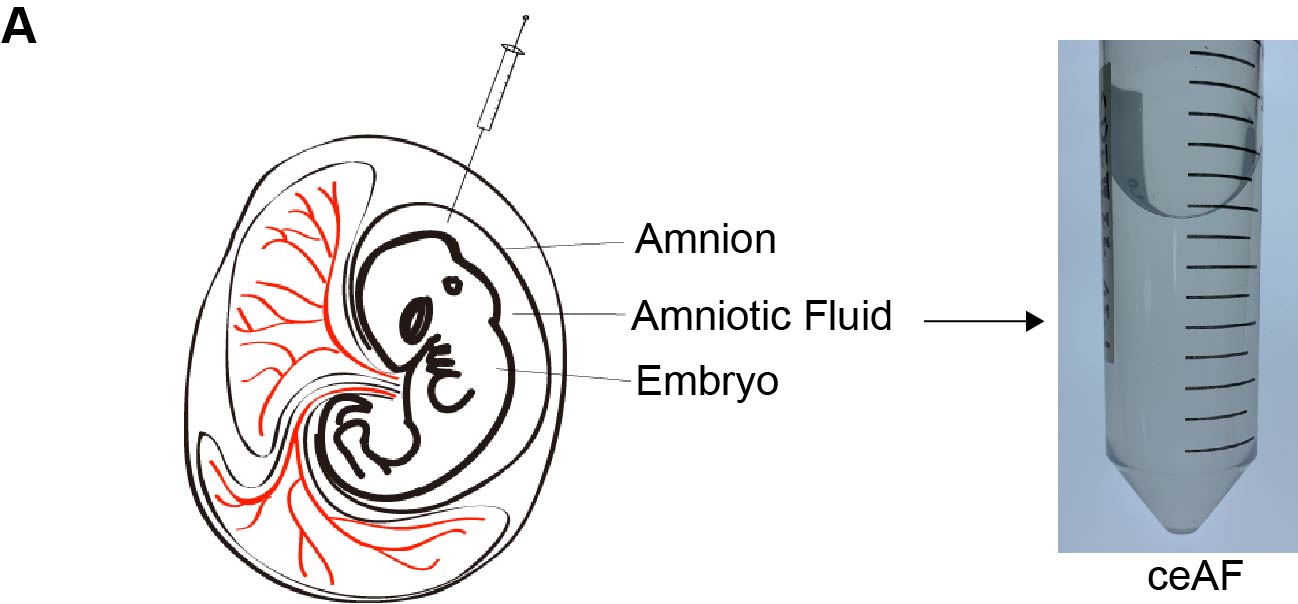
**

Supplemental Fig. 1 Schematic diagram of Chick Early Amniotic Fluid (ceAF) extraction.

**
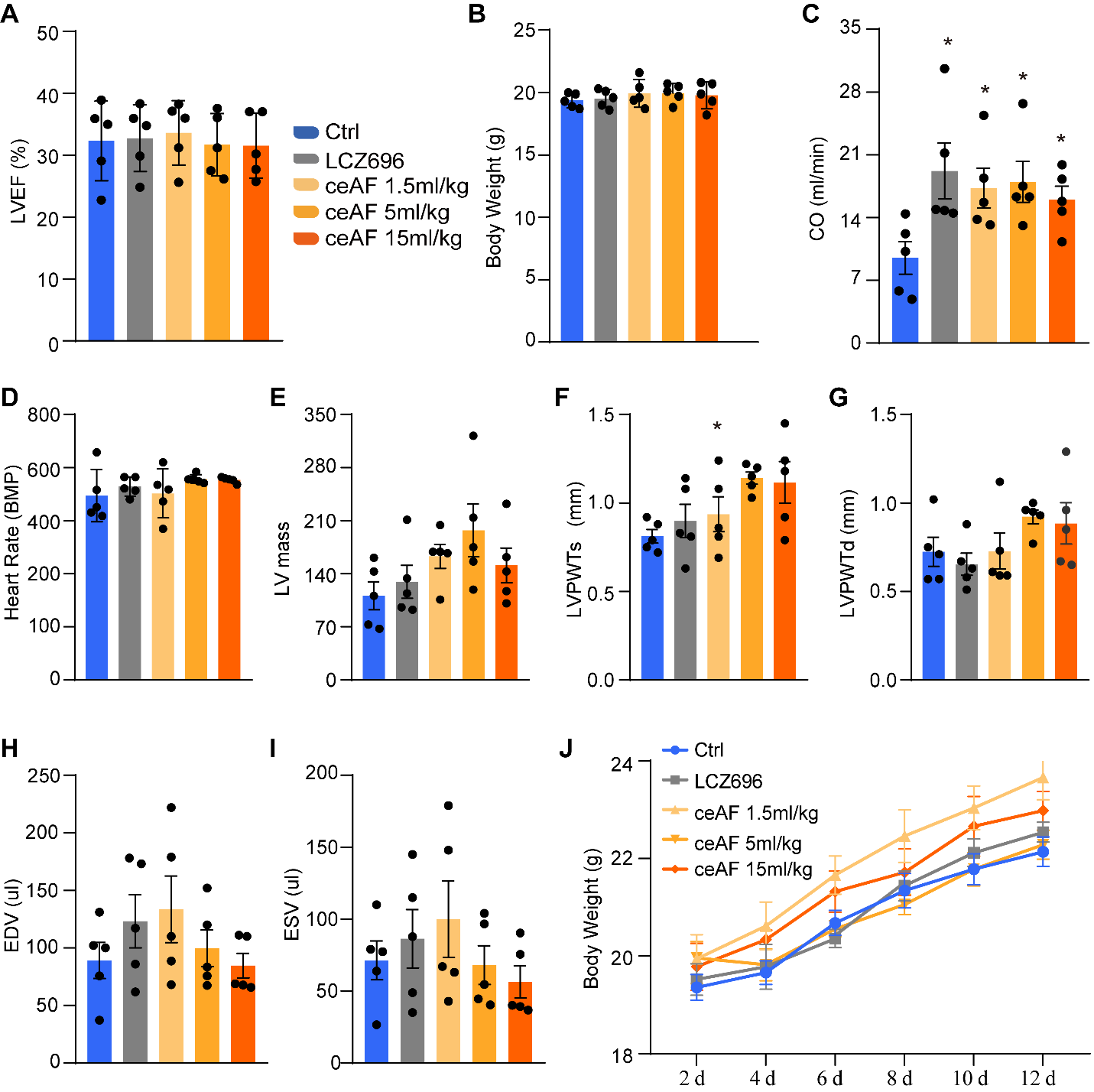
**

Supplemental Fig. 2 Analyses of cardiac function parameters before and after treatment of ceAF on mice MI model. **a-b** Grouping of mice based on the EF (**a**) and body weight (**b**) values on the second day after MI. Each group (n = 5) showed a very close average value of EF and body weight at this time. **c-i** Assessment of cardiac output (**c**), heart rate (**d**), LV mass (**e**), systolic left ventricular posterior wall thickness (LVPWTs) (**f**), diastolic left ventricular posterior wall thickness (LVPWTd) (**g**), end-diastolic volume (EDV) (**h**), and end-systolic volume (ESV) (**i**) of MI mice at day 13 after MI (n = 5. One-way ANOVA). **j** Average body weight values of MI mice from each group during the experiment. Data were mean ± SEM, *p < 0.05. Student’s two-sided t-test was used.

**
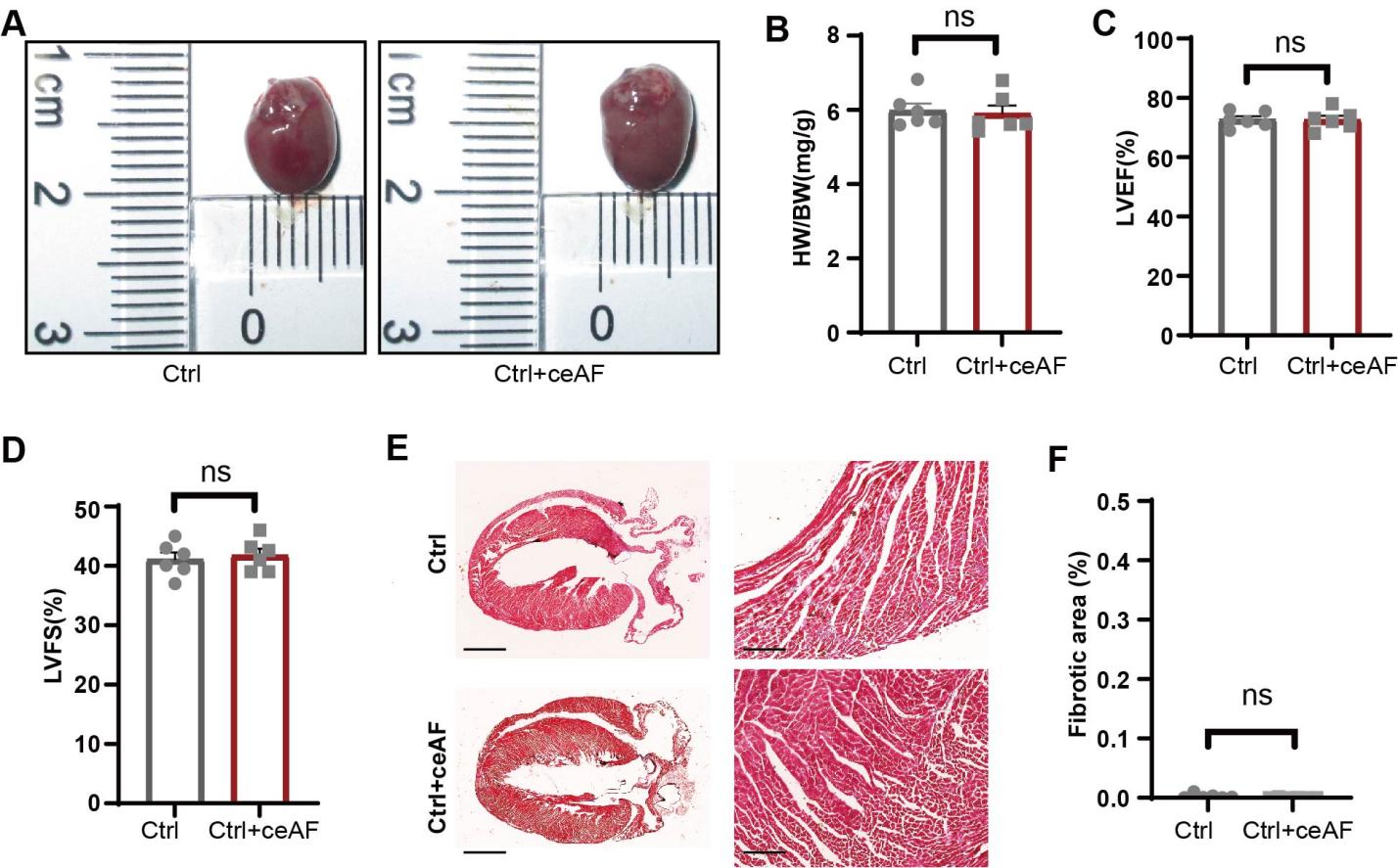
**

Supplemental Fig. 3 ceAF has no significant effect on normal heart function. **a** Macroscopic view of the heart of C57BL/6J mice treated with or without ceAF. **b** Quantification of heart weight/body weight ratio in each group, n=6. The LVEF (**c**) and LVFS (**d**) values in normal C57BL/6J mice treated with ceAF (5.0 ml/kg) (Ctrl+ceAF) or with 5% glucose (Ctrl) for 14 days. Data were mean ± SEM. **e** Representative Masson’s trichrome staining images of heart sections (left, scale bars: 2 mm) and high-magnification views of LV wall (right, scale bars: 50 μm) of each group. **f** Quantification of fibrosis area relative to myocardium in Masson’s trichrome staining sections. Data are mean ± SEM. ns, non-significant. n=6.

**
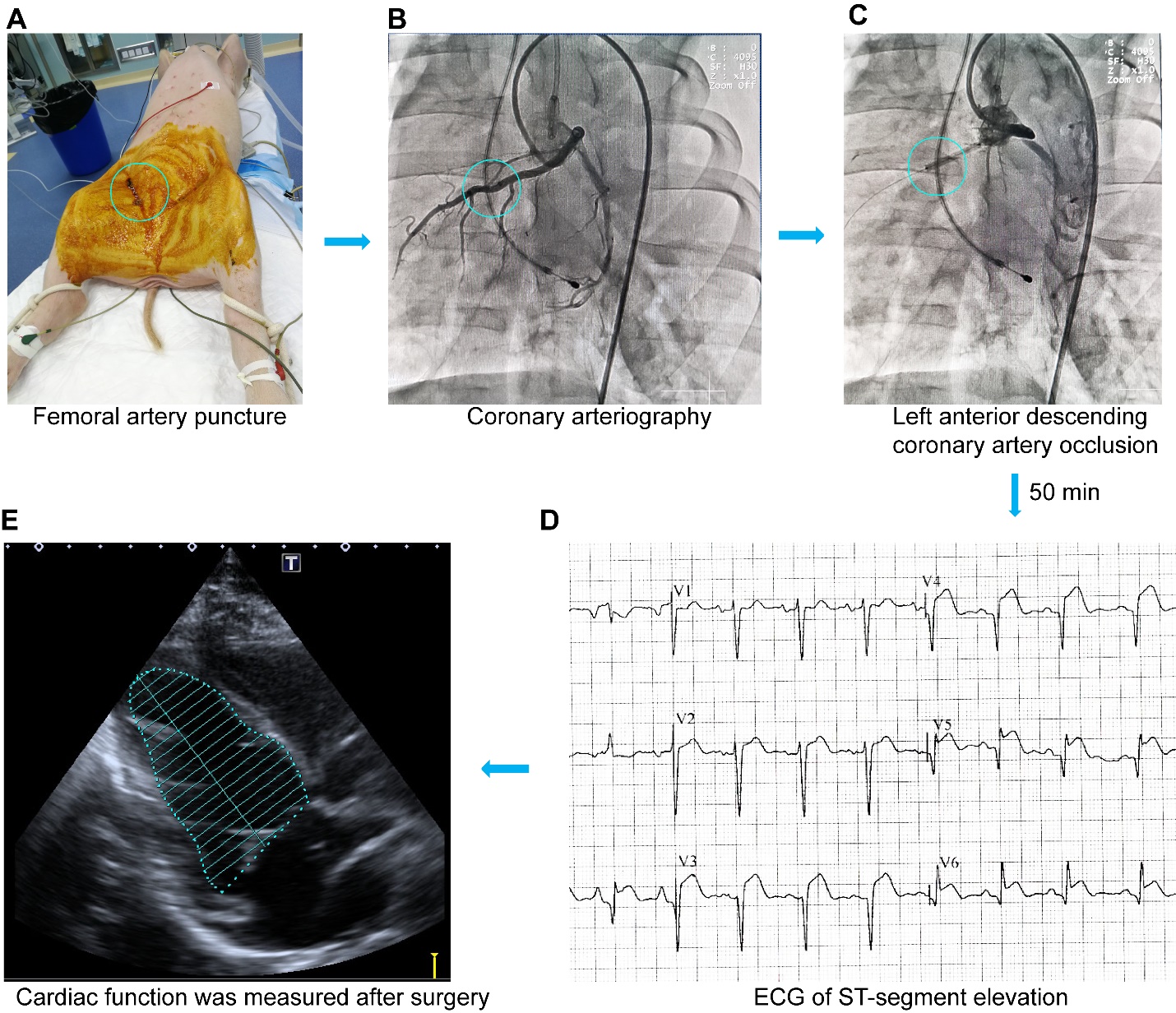
**

Supplemental Fig. 4 Establishment of ischemia reperfusion model in pigs. **a** Pigs were disinfected with iodide at the site of femoral vein puncture after anesthesia. **b** Coronary angiography was performed using contrast guidewire and the balloon was delivered to the left coronary artery. **c** LAD-D1 was selected as the infarct site. **d** The electrocardiogram V1 to V6 leads showed an electrocardiogram with T wave elevation. T wave elevation was documented in all pigs with IR. **e** Immediately after surgery, cardiac ultrasound was performed to measure cardiac function.

**
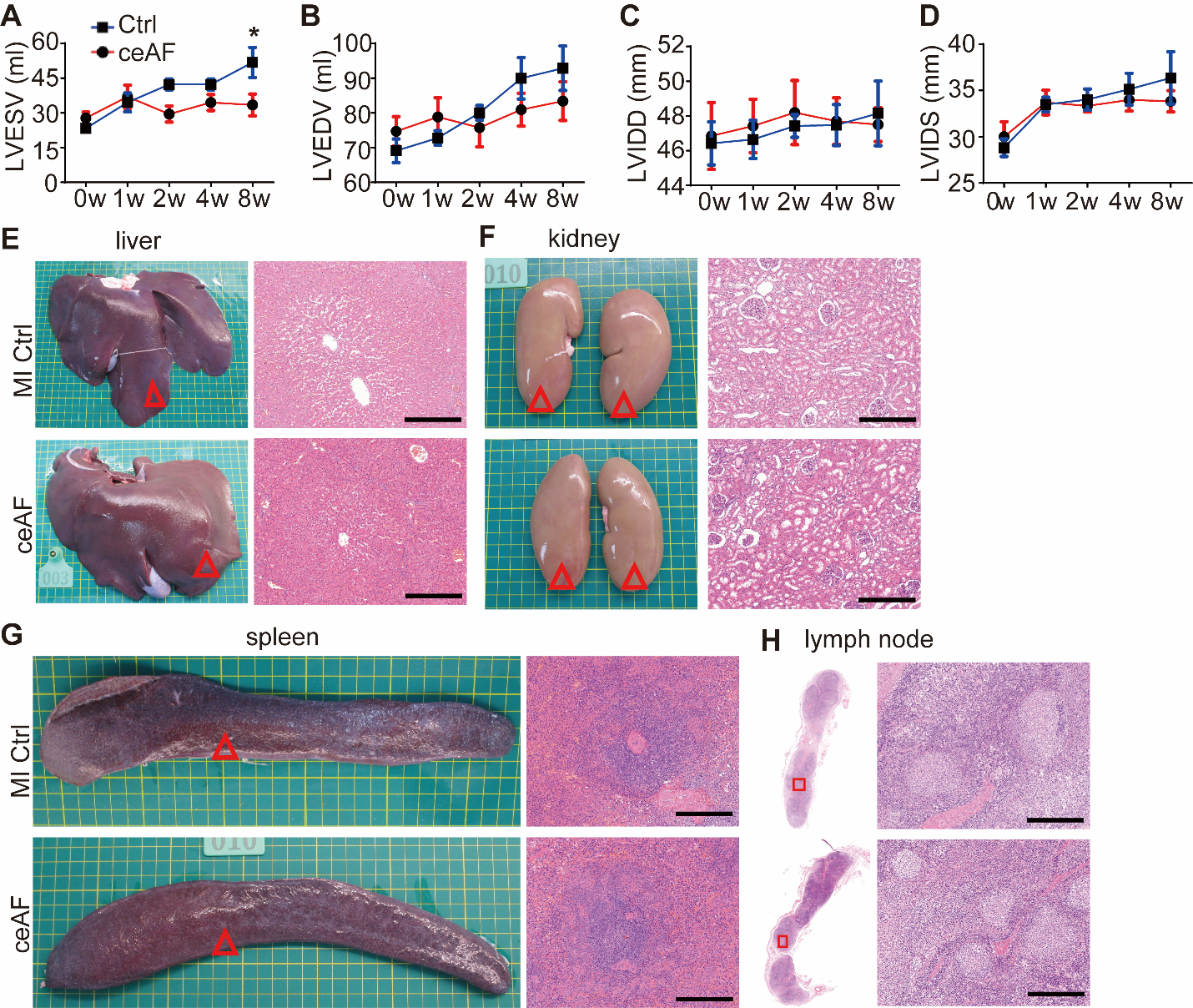
**

Supplemental Fig. 5 Additional cardiac function parameters and typical macroscopic and histological appearance of liver, kidney, spleen, and lymph node of MI pigs treated with ceAF after 8 weeks. Compared with those of the control group, ceAF-treated animals presented with a trend toward reduced LV end-systolic volume (LVESV) (**a**), LV end-diastolic volume (LVEDV) (**b**), LVIDD (**c**) and LVIDS (**d**). **e-h** Toxicity of ceAF treatment for 8 weeks. There were no obvious differences observed in all organs examined including liver (**e**), kidney (**f**), spleen (**g**), and lymph node (**h**) in IR pigs compared with Ctrl IR pigs treated with glucose solution (n = 4 for Ctrl, n = 5 for ceAF treatment).


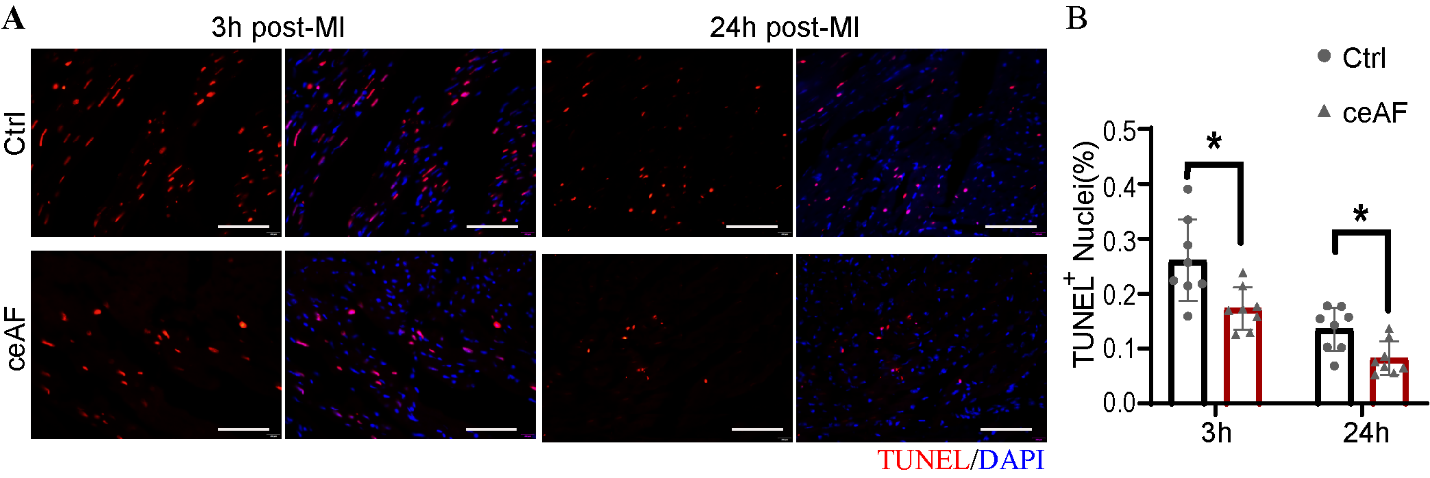


Supplemental Fig. 6 ceAF protects mouse cardiomyocytes from ischemia-induced apoptosis. **a** TUNEL staining of heart tissues in the border zone of infarction 3 hours and 24 hours after MI. **b** Percent TUNEL+ nuclei in mouse heart sections after 3h or 24h with or without ceAF treatment (n ≥ 3 mice), *, p<0.05. Scale bars, 100 mm.
